# Exploring Agricultural Livelihood Transitions with an Agent-Based Virtual Laboratory: Global Forces to Local Decision-Making

**DOI:** 10.1371/journal.pone.0073241

**Published:** 2013-09-05

**Authors:** Nicholas R. Magliocca, Daniel G. Brown, Erle C. Ellis

**Affiliations:** 1 Department of Geography and Environmental Systems, University of Maryland, Baltimore County, Baltimore, Maryland, United States of America; 2 School of Natural Resources and Environment, University of Michigan, Ann Arbor, Michigan, United States of America; University of Gävle, Sweden

## Abstract

Rural populations are undergoing rapid changes in both their livelihoods and land uses, with associated impacts on ecosystems, global biogeochemistry, and climate change. A primary challenge is, thus, to explain these shifts in terms of the actors and processes operating within a variety of land systems in order to understand how land users might respond locally to future changes in broader-scale environmental and economic conditions. Using ‘induced intensification’ theory as a benchmark, we develop a generalized agent-based model to investigate mechanistic explanations of relationships between agricultural intensity and population density, environmental suitability, and market influence. Land-use and livelihood decisions modeled from basic micro-economic theories generated spatial and temporal patterns of agricultural intensification consistent with predictions of induced intensification theory. Further, agent actions in response to conditions beyond those described by induced intensification theory were explored, revealing that interactions among environmental constraints, population pressure, and market influence may produce transitions to multiple livelihood regimes of varying market integration. The result is new hypotheses that could modify and enrich understanding of the classic relationship between agricultural intensity and population density. The strength of this agent-based model and the experimental results is the generalized form of the decision-making processes underlying land-use and livelihood transitions, creating the prospect of a virtual laboratory for systematically generating hypotheses of how agent decisions and interactions relate to observed land-use and livelihood patterns across diverse land systems.

## Introduction

Land-use change and its effects on land cover are major contributors to global environmental change [1]–[4]. Agricultural land-use is the most extensive and environmentally consequential land-use on Earth [5], [6]. Until recently (i.e., until the twentieth century), agriculture in most regions has been managed by smallholders: rural households with small amounts of land, who produce at least some of their own subsistence using family labor, and are only partially integrated into markets that are often inefficient or incomplete [7]–[9]. Smallholders remain important agents of land change [10], as their land-use practices have consequences for local food security, environmental sustainability, and economic development [11]. As economic globalization produces more and stronger teleconnections between urban and rural land systems, local land-use change is increasingly influenced by transitions from subsistence to commercial agriculture and non-farm livelihoods in response to changing regional/global market forces [12], [13].

Current understanding of the dynamics of agricultural land-use by smallholders has been built in part from a rich case-study literature related to ‘induced intensification’ theory [14]. Agricultural intensification was first described by Boserup [15] and Chayanov [16] as a process through which smallholders were forced to increase the labor intensity of cultivation through techno-managerial innovations to meet increasing production demands from rising population density. Case studies from a wide range of disciplines expanded on these insights to consider the roles of environmental suitability [17] and commercial agricultural activities [18]–[20] in driving agricultural intensification, which became more broadly labeled as ‘induced intensification’ theory [14]. Empirical support for this theory has been established by strong, positive correlations between agricultural intensity and population density, with environmental and economic pressures as mediating factors [14], [17], [21]. However, such methods cannot provide direct, mechanistic explanations of how smallholder behavioral responses to these factors lead to observed patterns of land-use and -cove change (LUCC). Furthermore, empirical evidence for the linkages between livelihood strategies and land-use patterns is based on fragmented literatures of local case studies [22], and is thus subject to the ‘one place, one time’ syndrome [23]. While synthesis methods, such as meta-analysis, can identify common patterns across empirical case studies, they cannot provide mechanistic explanations of how such empirical patterns emerge from underlying processes. Thus, current methods are insufficient for explaining the processes through which agricultural intensification arises within and across sites, and thus lack the means to form hypotheses about how patterns of LUCC result from the responses of actors to a range of global to local conditions.

Rindfuss and colleagues [24] propose that structured cross-site comparisons and syntheses can be facilitated by simulation models of LUCC, and agent-based models (ABMs) in particular because of their explicit representation of human decision-making processes. Parker and colleagues [25] attempted such a cross-site comparison with a set of ABMs of land-use change in frontier regions, but found that the same processes were often represented in different ways across models, which prohibited a clear synthesis of findings across studies. Here, we use a generalized ABM of land-use and livelihood decision-making as a virtual laboratory to investigate relationships between variations in agricultural intensity and the contexts within which agent decision-making occurs. The model’s simple structure links agents’ livelihood decisions to resulting land-use changes and enables generation of hypotheses about how land-use patterns are affected by varying local and global economic, environmental, and demographic conditions. Such a generative modeling tool, employed to productive effect in other fields [26]–[29], can be used to systematically explore the implications of agent decision-making for land-use and livelihood outcomes, as well as formulate new hypotheses that could lead to more nuanced theoretical explanations of land change processes than the inductive methods common in land change science [30].

We first ground the generalized ABM to induced intensification theory by evaluating outcomes against the empirical relationships derived in Turner and colleagues’ [17] meta-analysis of agricultural intensification and agent-level behavioral patterns described in the livelihoods and development literatures. Our aim is to verify that agents’ assumed decision-making models respond to changing economic, environmental, and demographic forces in realistic ways subject to heterogeneous risk preferences and environmental endowments. We then use a virtual laboratory approach to explore how livelihood strategies and land-use outcomes vary under a wide range of environmental and social conditions that are impossible to control for and observe in the field. As a result of this analysis we identify the effects of increased market integration on land-use and livelihood transitions, beyond those observed in the earlier work on induced intensification, as an area for further empirical testing.

## Methods

General characteristics of the model relevant for discussion of results are presented below. Detailed model description using a complete Overview, Design concepts, and Details (ODD) Protocol is provided in File S1. The ODD protocol is an accepted standard for presenting ABMs (http://www.openabm.org/page/standards), which describes the model’s purpose, design, and process overview and scheduling.

### 2.1. Landscapes

**Figure 1:**
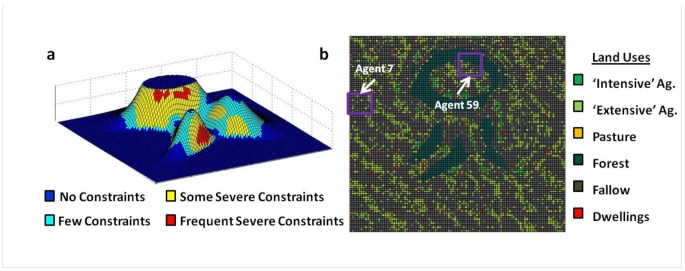
Hypothetical landscapes. Components of the hypothetical landscapes: (a) artificial topography and land suitability for agriculture; b) simulated land uses with agents 7 and 59 indicated (Section SI-5).

A stylized landscape of 100 by 100 square grid of cells (Fig. 1a) is created in MATALB with each cell representing one hectare (total area = 100 km^2^). An artificial landscape is used so that: 1) the full range of land suitability classes defined by GAEZ [31], [32], can be simulated within the same landscape; 2) the landscape does not represent any particular place and is thus generic; and 3) the landscape can be easily implemented and reproduced within any simulation environment. Turner and colleagues [17] found that the relationship between population density and agricultural intensity was dampened in favorable agricultural conditions, but exacerbated when significant constraints on agricultural productivity were present. Here, land suitability for agriculture is represented by a combination of slope and precipitation constraints. Slope is a proxy for soil suitability for agriculture with reductions in potential agricultural yields based on slope constraint classes [31]. Precipitation constraints are related to the length of the growing season [32], which impose additional reductions in potential agricultural yields evenly across the entire landscape. Experimental variations in agricultural suitability, which are a combination of slope and precipitation constraints, are implemented by turning slope and/or precipitation constraints ‘on’ or ‘off’ to investigate the influence of environmental conditions on agricultural intensity.

Six different land uses are represented as the outcomes of agent choices (Fig. 1b): three productive uses (intensive agriculture, extensive agriculture, and pasture for grazing livestock) and three non-productive uses (forest, fallow, and dwellings). Productive land uses are defined by functional group, rather than particular types (e.g., intensive or extensive versus irrigated rice or shifting cultivation based on cassava), and vary in their potential productivity, degradation/regeneration rates (Table 1), and labor costs (Table S1). Biophysical processes of primary production, land degradation, regeneration, and succession are represented using a simplified set of rules. ‘Intensive agriculture’ is defined as cultivation that uses external inputs (i.e., fertilizer, irrigation, and/or land improvement) to maintain productivity under repeated annual cultivation. ‘Extensive agriculture’ is defined as cultivation with no external input, and is therefore subject to land degradation under repeated cultivation. ‘Pasture’ represents rangeland on which livestock grazing occurs, and is subject to degradation if grazed repeatedly without fallow. Yields, which are reported in grain equivalents, are endogenously determined subject to environmental constraints of agricultural productivity and agents’ land-management actions. Labor costs of each land-use activity are represented relative to one another rather than to absolute values observed within any particular land system. Labor costs are expressed in terms of person-weeks per hectare per year to convert from or maintain a particular land use to another (Table S1), [41], [42].

**Table 1:** Model parameters for biophysical processes and agent attributes.

BIOPHYSICAL PROCESSES					
Land-Use		Avg. Yield^a^ (kg ha^−1^)		Degradation/RegenerationRates (kg ha^−1^ yr^−1^)	Avg. Price^b^ ($ kg^−1^) [33]
Intensive Ag. [34]		3,500		0/0	0.13
Extensive Ag. [35]–[37]		2,700		0.25/0.04	0.13
Pasture [38], [39]		1,700		0.18/0.5	0.13
**AGENT ATTRIBUTES**					
**Parameter Name**		**Value**	**Units**	**Notes**	**Source**
Household size	*h*	4	people	While the household size is held constant,the number of households per agentchanges with population density.	[40]
Total available labor	*L_tot_*	96	person-weeks household^−1^	Fifty-two weeks in a year less 15 for leisure,multiplied by 2 for 2 adultsand 1 for 2 children.	[41]
Minimum subsistence requirements	*δ_min_*	860	kg yr^−1^ person^−1^	Based on a diet of moderate meatconsumption; includes grain for feed.	[40]
Risk preferences	*R_pref_*	0 to 1	N/A	Certainty equivalent of a riskyactivity (i.e. risk-aversion).	[7], [8]
Initial household subsistencestock	*S_sub_*	2,580	kg	Initial food stocks are assumed tocover a year’s subsistence requirements.	N/A
Initial household money stock	*S_mon_*	1,426	dollars	Combined farm input costs and the costof a year’s subsistence needs at thelong-term average crop price witha market influence of 0.5.	N/A

a All agricultural yields are reported in grain equivalents.

b Agricultural product prices are assumed equal to control for agricultural commodity-differentiated price effects, and are based on the 5-year average farm price of wheat.

Using indicators of travel time to regional markets and purchasing power parity, the influence of regional and global market forces on local processes of LUCC is approximated with a global, normalized market influence (MI) index ranging from 0 to 1 [43]. Local farm gate and food prices, as well as monetary farm input costs and transaction costs associated with locating, securing, and maintaining non-farm wage employment, vary with market influence according to a set of hypothesized cost and price functions, [44]. These functions link global market influence index values to local farm-gate and food prices, farm input costs, and non-farm wages and transaction costs. Global commodity prices and U.S. minimum wage represent agricultural commodity prices and non-farm wages realized by a farmer in locations with a market influence index at or near one. Local product and factor prices and costs in locations with market influence less than one vary according to the functions provided in Section S1.2.4.

### 2.2. Agents

The model is designed in a way that addresses scaling and implementation challenges for large systems, and therefore uses agents to represent aggregates of multiple actors organized within settlements. Each agent represents a collection of smallholder households, the number of which varies with simulated population density, located in a single settlement that has 100 ha of land available for cultivation and settlement. Agent attributes are described in Table 1. Though most of the theory we draw upon conceives of the relevant decision making at the household level, a model of settlement agents is a reasonable approximation of the household context under the following assumptions: 1) land-use choices are significantly constrained by land suitability; 2) households in the settlement are equally endowed with labor, land, and capital; 3) settlement agents do not interact with one another; and 4) there are no significant spatial arrangements or interactions within the settlement that affect access to land. If all assumptions hold, a model of household agents would produce identical results in terms of the areas allocated to each land-use activity, though the spatial patterns may be different. In addition to facilitating model scaling, the settlement agent simplification permits population densities and land per capita to be easily manipulated, which is critical to the model experiments. This formalization also does not require detailed knowledge of local land allocation mechanisms, thus maintaining the generality of model outcomes. Indeed, one or more of these assumptions are likely to be violated in real land-use systems, and the implications of the settlement area simplification will be discussed in light of the results obtained.

**Figure 2:**
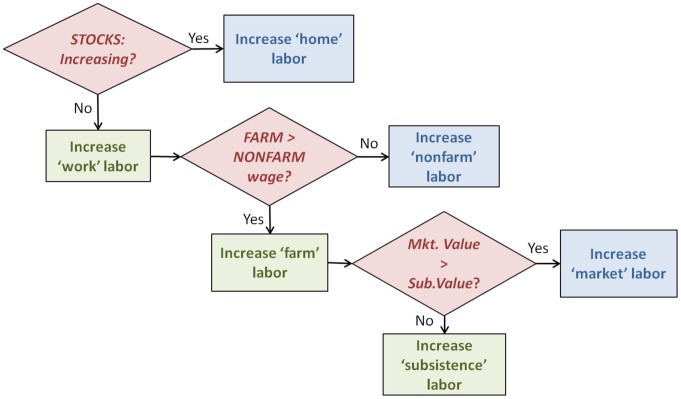
Labor allocation process. Heuristic decision tree of agents’ labor allocation process.

Agents’ behavioral rules are derived from smallholder household economic theories [7], [8], [15], such that labor and risk in land-use and livelihood activities are minimized. Each agent’s total labor is allocated between home, farm, and non-farm activities depending on the state of an agent’s food and income stocks (Fig. 2). Degree of market participation depends on the relationships between their perceived value of each activity subject to heterogeneous individual risk tolerances and exogenous farm-gate and food prices. According to empirical work by de Janvry and colleagues [45], consumption and production decisions are structured by differences between internal value of agricultural products (i.e., shadow price) and external price points. An agent’s shadow price is determined by the expected returns of agricultural production net of production costs. If an agent’s shadow price is greater than farm-gate prices, which are dependent on local market influence (i.e., both physical access to markets and purchasing power), but still lower than the costs of purchasing food on the market, then the agent is operating in a ‘price gap’ in which no market transactions take place and subsistence agricultural production will be the most favorable option [46]. If agent’s shadow price falls below both farm-gate and food prices, a mix of subsistence- and market-oriented agricultural production will likely produce the highest utility (subject to individual risk preferences). If the shadow price falls below the farm-gate price, but above food prices, then the agent will likely sell their agricultural production and purchase food on the market. Additionally, access to non-farm wage opportunities influences the intensity of land-use, as non-land-based income sources can supplement or fulfill food and income requirements and reduce labor to on-farm activities [7], [46]–[48].

The expected value of agricultural production is determined using a bounded rationality framework. Agents form expectations of agricultural yields and prices using a set of ‘backward-looking’ expectation models that extrapolate past trends one period into the future. The expected utilities of each activity are calculated simultaneously based on both the marginal production (agricultural or monetary) per unit labor costs (time or monetary) and maximization of production net of labor costs (i.e., profit-maximization). Expected pay-offs for subsistence-oriented activities are based on the former utility model, whereas expected pay-offs for market-oriented activities are calculated using the latter model. Agents select the best land-uses and livelihood activities based on these expected utilities, and can adapt to changing market opportunities or declining/improving yields by alternating between decision models and/or production modes (i.e., alternative land-uses or subsistence- versus market-oriented modes). A more detailed description of and equations for agents’ decision models are provided in Sections S1.4.1 and S1.4.6. While agents’ decision models are prescribed according to theory, agents’ choices among a set of possible livelihood activities in response to changing conditions are subject to individual risk preferences and learning and are thus emergent.

### 2.3. Simulation

Parameter testing and selection is based on the pattern-oriented modeling (POM) approach [49], [50].The main principle of POM lies in the reproduction of multiple patterns observed in real systems simultaneously. If a model can accomplish this, one can conclude that the model’s process representation and internal structure are reasonably consistent with those of the real system [49], [50]. Three target patterns are identified from the economic development and livelihoods case-study literatures: 1) the presence of a ‘normal surplus’ in agricultural production, 2) meeting or exceeding minimum aspiration levels, and 3) ‘consumption smoothing’. These patterns represent ‘stylized facts’ describing empirical regularities in agent-level behaviors associated with land-use and livelihood decisions [9], [45]. *Normal surplus* is a level of agricultural production commonly observed in smallholder farming systems. In an ‘ideal’ subsistence system (i.e. low market influence), production constraints and uncertainty in crop yields lead smallholding farmers to minimize risk of and labor in production by producing only as much as is needed to meet subsistence needs (i.e. little or no surplus, termed ‘normal surplus’) [9]. *Minimum aspiration level*, in this context, is defined as the minimum income needed to support farming activities and/or purchase food on the market. As market influence increases, social structure and aspirations change and transform behavior [9]. Consequently, production levels exceed what is necessary to meet subsistence needs, as surplus can be sold on the market, and labor is allocated increasingly to maximize profits from market crops. *Consumption smoothing* is frequently observed in smallholder consumption patterns [7], [45], [46], and is measured here as the coefficient of variation in the difference over time between agricultural production and monetary income levels relative to subsistence needs. Table S2 provides the specific threshold values used to implement each of these target patterns.

Simulations proceed in MATLAB as follows. Landscape outcomes are modeled as a result of the livelihood and land-use decisions of agents in annual increments over a twenty-year period (with the first ten as model spin-up). The landscape is initialized with land uses according to agricultural suitability. Total subsistence requirements, available labor, and initial stocks are allocated to each agent based on population density. Agents form location-specific risk-weighted expected returns for all possible land uses in both subsistence and market production on their land according to the logic described above. For all possible land uses in each of the cells of an agents’ landholdings, expected marginal return on labor from subsistence production and expected profits from market production are calculated to obtain expected marginal utility. Agents first allocate subsistence labor to cells that maximize marginal expected utility from subsistence production until subsistence labor or land constraints are met. Market labor is then allocated to remaining cells that maximize expected marginal utility from market production until market labor or land constraints are met. Land uses are chosen for each cell and actual returns net own consumption/input costs are calculated. Food and monetary stocks are updated, and price and yield expectations are formed for next period. Rates of labor re-allocation between activities in the next period depend on the direction and magnitude of changes in food and monetary stocks. Landscape cells are updated and degrade/regenerate with current land uses. An overview diagram of model processes and scheduling is provided in Figure S2.

### 2.4. Model Experiments

Using a virtual laboratory approach, population density, market influence, and environmental constraints were systematically varied to explore the role each factor played in shaping livelihood strategies and land-use outcomes. Population densities were varied from 16 to 144 people km^−2^, which characterize extensive to intensive cultivation systems, respectively, as predicted by induced intensification theory [17]. Market settings were varied with index values of MI ranging from 0.2 to 0.8, such that the lower end of the range represented levels of market access in which dominantly subsistence-oriented land-use systems would be expected [17], whereas increasingly market-oriented land-use systems would be expected towards the upper end of this range. Experimental landscapes (Table 2) were created by turning slope and/or precipitation constraints ‘on’ or ‘off’ to explore the mediating effects of land suitability on land-use intensity and livelihood strategies.

**Table 2:** Experimental landscape settings.

Experimental Landscape^a^	Brief description
*Baseline*	Reduction in potential agricultural productivity due to slope constraints according to topography, and a 50 percent reduction due to precipitation constraints.
*Slope-Only Constrained*	Reduction in potential agricultural productivity due only to slope constraints according to topography.
*Precipitation-Only Constrained*	Reduction in potential agricultural productivity of 50 percent due only to precipitation constraints.
*Neutral*	No reductions in potential agricultural productivity.

a Experimental landscapes are created by turning ‘on’ or ‘off’ slope and/or precipitation constraints.

A total of 100 different experimental combinations were investigated across five population densities, five market influence settings, and four different experimental landscapes. For each combination of population, market, and environmental settings, the model was run up to 60 times depending on the number of cost and price function parameter sets that produced ‘successful’ outcomes. A model outcome was deemed ‘successful’ when all three agent-level behavioral patterns are reproduced simultaneously. Multiple plausible sets of cost function parameters were generated using a genetic algorithm to capture variations in local cost relationships for each MI index value and population density tested. Magliocca and Ellis [44] provide a detailed description of the procedure used to parameterize price and cost functions. Given the results of the genetic algorithm coupled with pattern-oriented modeling criteria, a total of 1,088 model runs were performed across all experimental settings.

### 2.5. Statistical Analyses

Differences in model runs were evaluated in terms of cropping frequency, a measure of agricultural intensity (calculated as the percent time a given cell was cultivated over the simulation period, [17]), percent of labor allocated to subsistence versus market oriented agriculture and wage labor, evenness of labor allocation across the different activities, and total agricultural production. Shifts in livelihood strategies, or ‘livelihood transitions’, were detected using a series of one-way Kruskal-Wallis tests comparing median values of each variable at consecutive pairs of market influence settings. Post-hoc analysis tests estimated differences between mean ranks at each market influence setting using the Scheffe S multiple comparison procedure [51], [52], to account for the effects of multiple comparisons across experimental settings. Statistically significant (Table 3) differences between mean group ranks were interpreted through the lens of induced intensification and rural livelihoods theories as indicating shifts between livelihood strategies.

**Table 3:** Statistics from the multiple comparison of key livelihood variables.

	Direction of Market Influence Index Comparisons^a^			
Variable	0.20 to 0.35	0.35 to 0.50	0.50 to 0.65	0.65 to 0.80
% Farm Labor	96.3 (−10.0–202.7)	209.7** (98.9–320.6)	240.4** (129.7–351.0)	187.5** (79.4–295.6)
% Market-Oriented Labor	225.7** (118.1–333.3)	112.9** (0.76–225.0)	130.1** (18.2–242.0)	58.1 (–51.2–167.5)
Surplus Ratio	210.1** (102.4–317.7)	124.9** (12.7–237.1)	166.9** (54.9–278.8)	82.0 (–27.4–191.4)
Evenness Index	217.6** (109.9–325.2)	203.0** (90.9–315.2)	120.2** (8.3–232. 2)	73.7 (–35.8–183.1)

** *p*< = 0.01

a Results from the multiple comparison procedures using one-way Kruskal-Wallis tests comparing each variable at consecutive pairs of market influence settings. The estimated difference (top) in and 99 percent confidence interval (bottom) of mean ranks at each market influence are shown. If the confidence interval does not contain zero, then the mean group ranks are significantly different at the 0.01 level.

## Results

### 3.1. Verification of Induced Intensification Predictions

**Figure 3:**
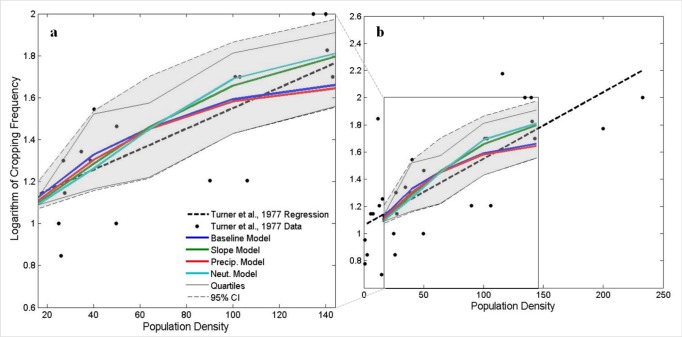
Cropping frequency versus population density. Relationships between population density and cropping frequency adapted from Turner et al. (1977) (black points and dashed regression line) within the settings investigated by the model (a) and the entire range of the Turner et al. (1977) data. These are compared with model outcomes for variations in environmental conditions represented by the experimental landscapes. Bold lines represent the median modeled cropping frequencies for each landscape. Thin black lines represent the first and third quartiles, and the gray shaded region marks the 95 percent confidence interval.

**Figure 4:**
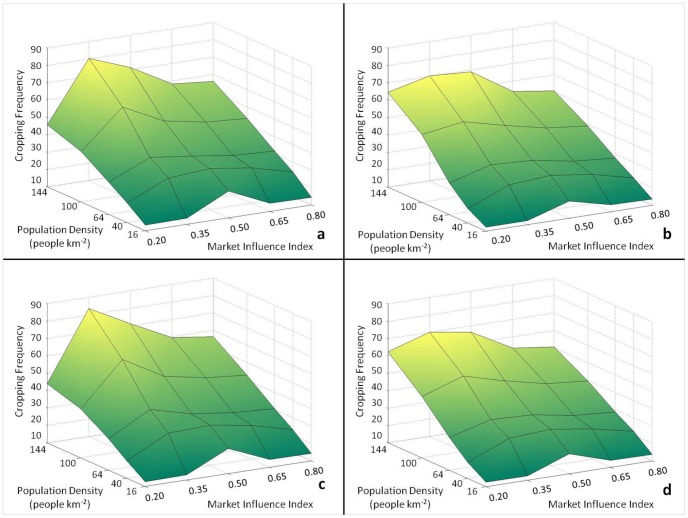
Cropping frequency across experimental settings. Cropping frequency in parameter spaces for a) baseline, b) neutral, c) only precipitation constrained, and d) only slope constrained landscapes.

Overall, the population-agricultural intensity relationship produced by this experiment was comparable to that observed by Turner and colleagues [17] across 29 tropical subsistence cultivator groups. Across experimental landscapes that varied in precipitation and slope, increases in population density produced increases in agricultural intensity as indicated by changes in cropping frequency (Fig. 3). Cropping frequencies were generally higher in landscapes subject to precipitation constraints on agricultural productivity (i.e. baseline and only precipitation constrained landscapes, Figs. 4a and 4c), while slightly lower in more favorable environments (i.e. ‘neutral’ and only slope-constrained landscapes, Figs. 4b and 4d). These results are consistent with findings that environmental extremes often exacerbate or dampen, respectively, the agricultural intensification process in response to population pressures on food demands [9]. Labor allocation and land-use decisions by two different agents (Fig. S1) illustrate the adoption of intensive cultivation in response to increased population pressure and declining yields per unit labor and land (Fig. S3).

**Figure 5:**
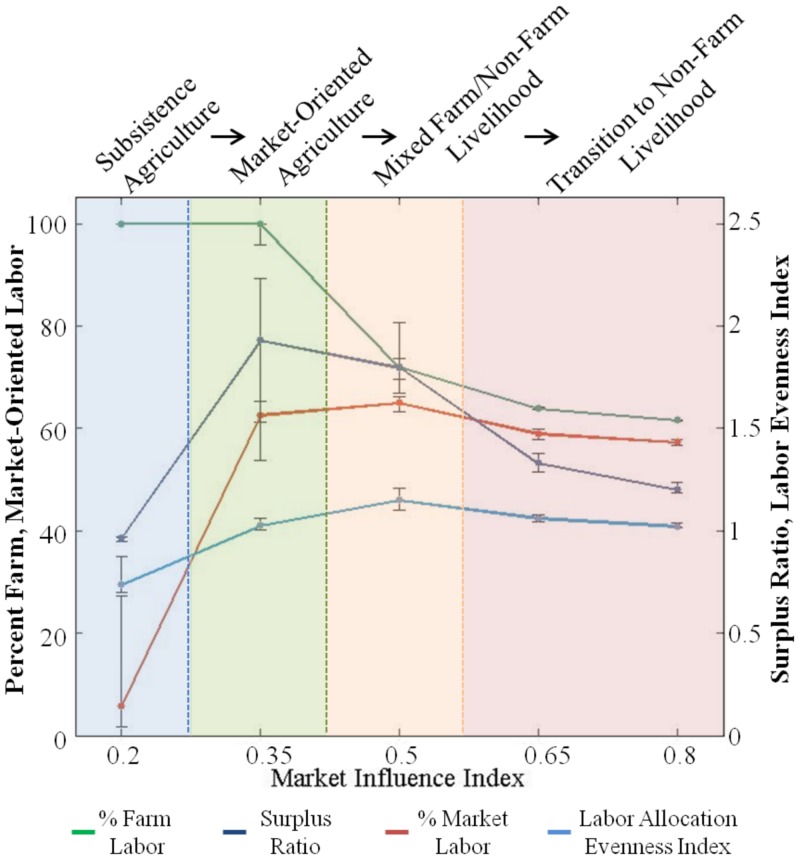
Transitions in livelihood strategies. Evidence for livelihood strategy transitions based on statistically significant changes in labor allocation and production variables across market influence settings. The dashed blue, green, and orange lines represent the first, second, and third ‘market transitions’ referred to in the text. Each ‘market transition’ is characterized by increased participation in the market through market-oriented livelihood activities (Fig. S5).

Variations in MI also resulted in significant changes in agricultural intensity. Figure 5 displays changes in key variables describing labor allocation (percent on-farm labor, percent market-oriented labor, and an index of the evenness of labor allocation across all activities) and levels of surplus agricultural production. At the lowest level of MI (0.2), agricultural production was mostly subsistence-oriented, which was demonstrated by a nearly 100 percent labor allocation to on-farm activities. Livelihood diversification, measured with an index of the evenness of labor allocation, was also relatively low reflecting poor access to markets and reliance on subsistence production. However, a slight increase in MI (0.35) precipitated a market-influence-driven ‘livelihood transition’ with increased market-oriented agricultural production. Significantly different farm and market-oriented labor allocation, livelihood diversity, and agricultural production levels were observed (Table 3). Market-oriented labor increased while farm labor remained high, which increased agricultural production levels and created a surplus. This transition also resulted in higher cropping frequencies regardless of population density and environmental conditions, as the percentage of labor allocated to on-farm and market-oriented activities increased. Within these general trends, individual agents’ responses to MI varied according to the productivity of their land and idiosyncratic risk preferences.

### 3.2. Land-use and Livelihood Outcomes from Model Experiments

**Figure 6:**
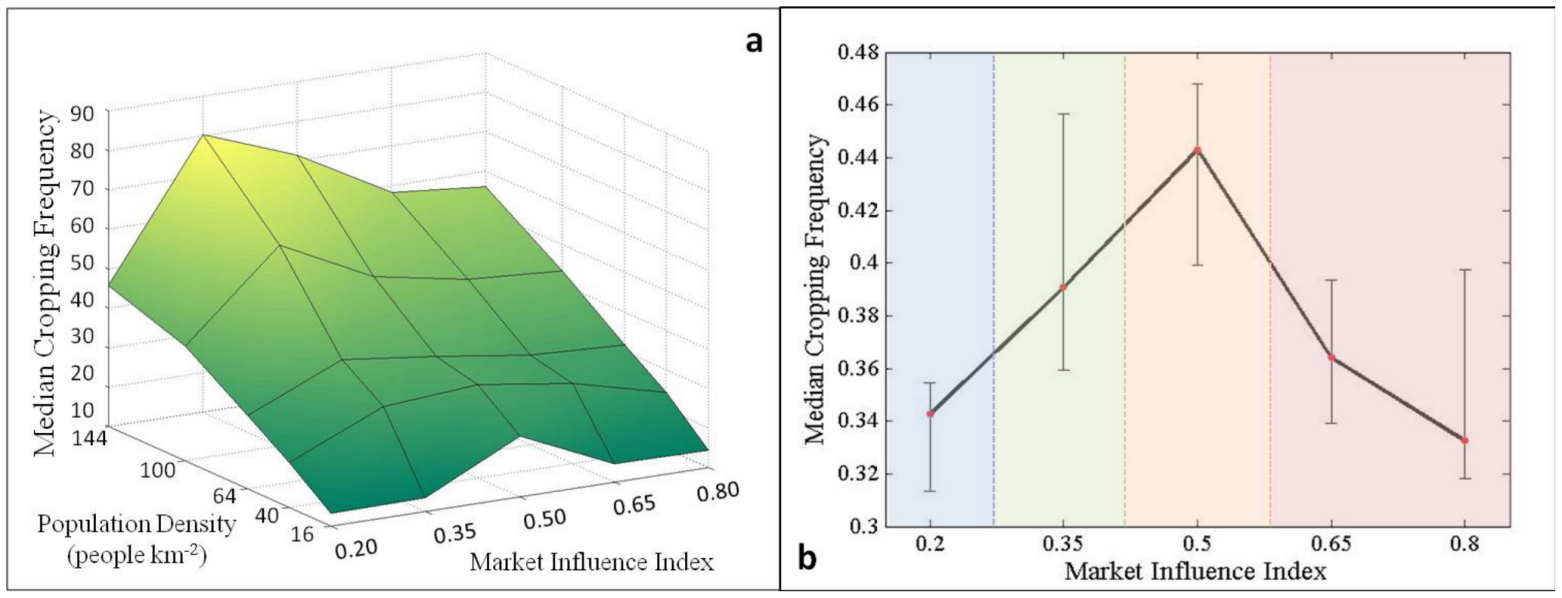
Influence of livelihood transitions on cropping frequency. (a) Variations in median cropping frequencies from the baseline landscape in response to population density and market influence index settings. (b) Changes in median cropping frequency in response to shifts in livelihood strategies are indicated by color-coded regions with non-overlapping confidence intervals.

As MI increased from 0.35 to 0.5, a second ‘livelihood transition’ to a regime in which labor was more evenly allocated between on-farm and non-farm livelihood activities occurred. The evenness of labor allocation among farm and non-farm activities reached its highest value (Fig. 5), which indicated the increased importance of livelihood diversification to include non-farm income sources. Improved access to non-farm wage opportunities with increasing MI resulted in increased labor allocation to non-farm wage activities while decreasing on-farm labor (Fig. 5), though no significant change in cropping frequencies was detected (Fig. 6). With a median value of above 70 percent, on-farm labor was still the dominant livelihood strategy, which kept agricultural intensity high. However, the variation around the observed median cropping frequency suggests that population density affected the MI at which the shift to a mixed farm and non-farm livelihood strategy occurred.

The level of MI at which this second ‘livelihood transition’ emerged varied according to environmental and land/labor constraints related to population density. Non-farm wage employment became an increasingly important livelihood activity at MI values of 0.35 to 0.50 for population densities of 40 people km^−2^ and greater, compared to MI values of 0.50 to 0.65 for a population density of 16 people km^−2^ (Fig. S4e). At higher population densities, land constraints required intensive cultivation methods to meet production demands, which had lower returns to labor than the extensive cultivation employed at lower population densities. While a combination of intensive and extensive cultivation methods could be used at 40 and 16 people km^−2^ to expand agricultural production for the market, non-farm wages became increasingly attractive relative to on-farm wages at higher population densities due to land constraints (Fig. S5).

The final ‘livelihood transition’ between 0.5 to 0.65 was characterized by a shift from a mixed farm and non-farm livelihood strategy to an increasingly non-farm strategy. The increased role of non-farm labor was evident from decreases in farm labor and agricultural surplus (Fig. 5), as well as cropping frequencies (Fig. 6). Diversity in livelihood activities declined as agents became more fully integrated into markets. Market forces, such as improved access to non-farm employment and relatively cheaper farm inputs due to lower transaction costs, became stronger drivers of livelihood decisions than population pressures or environmental conditions. Variations in labor allocation variables closely followed changing market conditions, which demonstrated the linkages between agents’ adaptive decision-making, land-use changes, and the emergence of ‘livelihood transitions’.

## Discussion

Idealized representations of smallholder land-use decision-making generated landscape-scale patterns of agricultural intensification consistent with those predicted by induced intensification theory (Fig. 3). More importantly, agents’ livelihood decisions in response to increased population pressure, market influence, and environmental constraints were linked to processes of land-use intensification. Hypothesized ‘livelihood transitions’ were observed in key variables measuring agents’ labor allocation and agricultural production decisions as market influence increased. Heterogeneity in the productivity of land and agent risk preferences also had a demonstrable influence on individual agents’ responses to increased population pressure and market influence (Fig. S1).

Extending the findings of Turner and colleagues [17], simulation results demonstrated the emergence of multiple potential ‘livelihood transitions’ through which market forces exert influence that extends beyond the classic relationship between agricultural intensity and population density. The first livelihood transition to intensive agricultural production for the market coincided with recent advances of induced intensification theory that consider the introduction of commercial production and increased market participation [20]. The second transition, to increasingly diversified livelihood activities, was consistent with the livelihoods case-study literature that finds diversification to be a frequent smallholder strategy to cope with inefficient or incomplete markets and/or resource scarcity [8], [45], [46]. The third transition resembled the effects of rural-to-urban migration and/or remittances in which land-use intensity declines [12] due to the growing percentage of rural household incomes from non-farm wage sources.

By representing land systems as open systems, we were able to connect local decision-making processes, environmental constraints, and land-use outcomes to global-scale forcings, such as markets and anthropogenic changes in precipitation [12]. Furthermore, the model was grounded to induced intensification theory at the decision-making level by comparing both agent-level behaviors and land-use outcomes to empirical regularities. Formal representation of the decision-making mechanisms assumed to drive agricultural intensification provided a means for generating hypothesized adaptive behaviors and mechanisms that can be tested against empirical data. Explicitly modeling agents’ land-use and livelihood decision-making processes allowed a direct connection to be made between the production intent of agents and more or less intensive land-use outcomes. Thus, clear linkages can be made between large-scale, exogenous environmental and market factors, internal smallholder production logic and livelihood strategies, and how interactions between those factors drive local land-use choices and the structure of land systems overall. The next step will be to systematically generate hypothetical transitions across a set of diverse land systems, which can then be tested against empirical case-study observations to search for patterns in the causes and consequence of land change across sites.

Several important model limitations and simplifications are worth highlighting. An important model limitation was the representation of a maximum cultivation intensity of single cropping without fallow annually, which does not capture more intense cultivation practices of multi- or inter-cropping annually. Thus, the model currently underestimates cropping frequencies observed by Turner et al. [17] at high population densities. In addition, representations of land productivity, risk preferences, labor and transaction costs, and market influence were all stylized. Empirical parameterization to a specific land system would yield quantitatively different results. However, because we aimed to reproduce relationships predicted by induced intensification theory and demonstrate its flexibility across a wide range of land systems, empirical realism was sacrificed for theoretical fidelity.

A major simplification was the representation of agents’ labor allocation and land-use decisions at the settlement level, rather than a spatially explicit, household-level representation. Such an approach allowed explicit linkage of varying demographic, economic, and environmental forces and heterogeneous decision-making specific to each land-use activity. Yet it did not require detailed knowledge of local land allocation mechanisms or social relations, thus simplifying model construction and maintaining the generality of model outcomes. A model in which individual households and land-tenure rules are explicitly represented would provide additional insights about the spatial patterns and scales of decision making, roles of institutions, and interactions among households through information, material, and capital flows. Indeed, this is a fruitful direction for further model development and the testing of hypotheses about the importance of household versus settlement-level decision-making and whether some land systems might be effectively represented by settlement agents versus those requiring a household-level representation.

Such additions, however, would reduce the simplicity, and therefore generality, of the model’s structure, and make the dynamics of interest between land per capita and agricultural intensification more difficult to interpret and implement broadly. Furthermore, for the purposes of quantifying patterns of global environmental change, explaining the amount of land change in a landscape in response to large-scale forces - for which the settlement agent representation is useful - may be more important than knowing the exact spatial arrangement of those changes. Used as an agent-based virtual laboratory, this framework enables systematic testing of demographic, economic, and environmental factors independent of local context-dependent social structures and institutions, as well as a generic formalization of global to local linkages facilitating model application and comparison across different land systems. Having demonstrated important mechanistic explanations for observed patterns of agricultural intensification, a next step will be to evaluate the hypothetical livelihood transitions generated by these modeling experiments against empirical data, and more broadly to test hypotheses about the influence of other potentially important processes on land system outcomes, including local social networks and institutions, across different land systems and locations.

The cumulative effects of local land-use change are regionally and globally pervasive. Accordingly, land-use change has been identified as a key component of global environmental change [1]–[4]. Yet our ability to scale-up knowledge of local land change processes to scales relevant to regional and national policy- and decision-making remains extremely limited [51], [52]. To address this shortcoming, simulation models that can produce realistic land change dynamics without over-specifying the processes involved to fit a particular local condition of land change are essential.

Certainly, over-simplifying the context in which land-use decision-making is embedded can lead to incomplete and/or incorrect understanding of the forces that shape land-use choices. On the other hand, representing the full complexity of social interactions that influence land-use choices runs counter to the aim of understanding larger-scale trends in land change; the impracticality of acquiring such detailed data across sites, coupled with the limitations of human cognition to navigate such complexity, is prohibitive. As we demonstrate here, the way forward in understanding land-use as a global change process will require starting with simple models, testing them theoretically and empirically, and gradually building-in more complexities through an experimental, virtual laboratory approach. Ultimately, this approach will form and test hypotheses about where and when additional complexities reflecting local context are and are not important for explaining land-use and livelihood patterns.

## Conclusions

Our modeling approach generates generalizable yet mechanistically rich descriptions of the agricultural intensification process that can be used to generate hypotheses of how and under what conditions the adaptive responses of land users to changing economic, environmental, and demographic forces can cause profound transitions in land systems. By explicitly representing land users’ decision-making processes and actions, we were able to explore mechanisms underlying potential livelihood transitions and their impacts on the landscape that arise from agents’ responses to a range of different local and broad-scale influences. Because it is generalized, and not tied to a specific natural or social context, our model represents a virtual laboratory capable of investigating how individual land-use and livelihood decisions are coupled to local and global pressures and outcomes. This approach offers new opportunities to generate and eventually test hypotheses of the importance of local- versus global-scale influences on land system outcomes across sites.

The rate and scale of land change driven by economic globalization has already surpassed the scope of conventional, location-based research for understanding local LUCC globally. Furthermore, teleconnections between urban and rural land-use systems link the demographic, economic, and environmental consequences of individual land users’ decisions to distant land systems and vice versa [13]. Thus, it is imperative to understand the underlying behavioral rationale of land-use decisions. The agent-based virtual laboratory approach enables experimental testing of hypotheses relating to the adaptive responses of local land-use decision-makers under changing large-scale driving forces, such as how agents’ motivations might change as economic globalization restructures local economic opportunities. A primary motivation for creating this generalized modeling framework is to be able to conduct systematic comparative studies on potential land change and resource use trajectories across different regions and land systems. Ultimately, this approach enables a more integrated and dynamic global understanding of anthropogenic land change processes, from which a more nuanced understanding of the global context and specific driving forces shaping particular regions is possible.

## Supporting Information

Figure S1 **Livelihood activities and resulting LUCC for agents 7 and 59 (as indicated on the map in** **Figure 1** **) with a market influence of 0.5 and population density of 64 people km^−2^.**
(TIF)

Figure S2 **Process overview and scheduling presented as pseudo-code.**
(TIF)

Figure S3 **Marginal agricultural yield (per unit labor time and land) in response to varied market influence and population density levels with baseline environmental conditions.**
(TIF)

Figure S4 **(a) Percent labor allocation toward market-oriented livelihood activities, (c) on-farm livelihood activities, and (d) non-farm livelihood activities in response to varied market influence and population density levels with baseline environmental conditions.** Changes in labor allocation in response to shifts between livelihood strategies indicated by color-coded regions to Figure 4 (b, d, and f).
(TIF)

Figure S5 **Percent intensive (a) and extensive (c) cultivation in response to variations in population density and market influence index settings from the baseline landscape.** (b and d) Changes in percent intensive and extensive cultivation in response to shifts in livelihood strategies are indicated by color-coded regions according to Figure 4.
(TIF)

Table S1 **Combined labor and input costs.**
(DOCX)

Table S2 **Performance criteria associated with three agent-level behavioral patterns used to implement the genetic algorithm.**
(DOCX)

File S1 **Supplementary Information.**
(DOCX)
