# Supplementary figures and images for: Exploring Agricultural Livelihood Transitions with an Agent-Based Virtual Laboratory: Global Forces to Local Decision-Making
Source: PLoS One. 2013 Sep 5;8(9):e73241. doi: 10.1371/journal.pone.0073241 (PMC3764159; doi:10.1371/journal.pone.0073241)

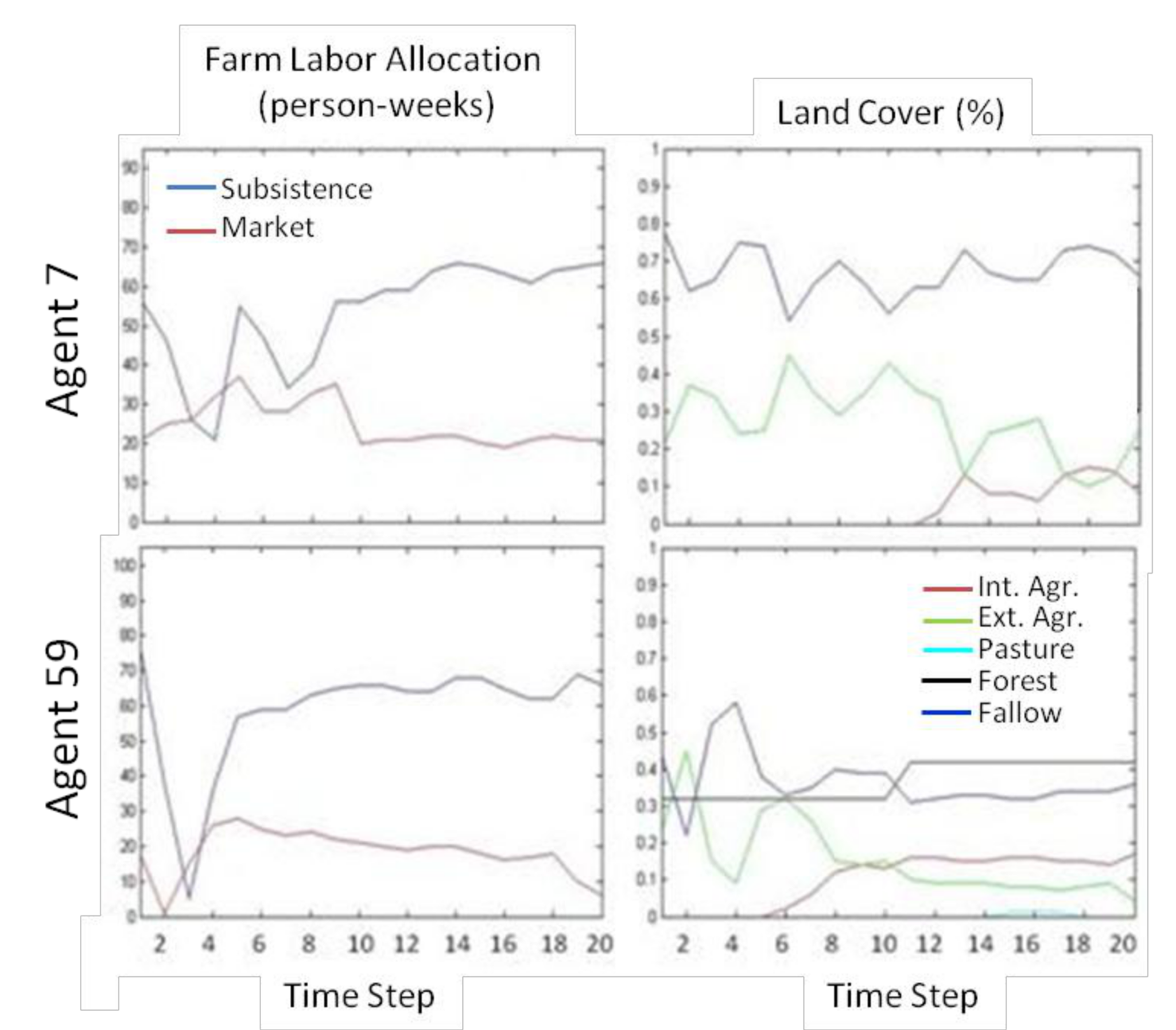

Supplement: Figure S1 — Livelihood activities and resulting LUCC for agents 7 and 59 (as indicated on the map in Figure 1 ) with a market influence of 0.5 and population density of 64 people km−2. (TIF) [file pone.0073241.s001.tif]

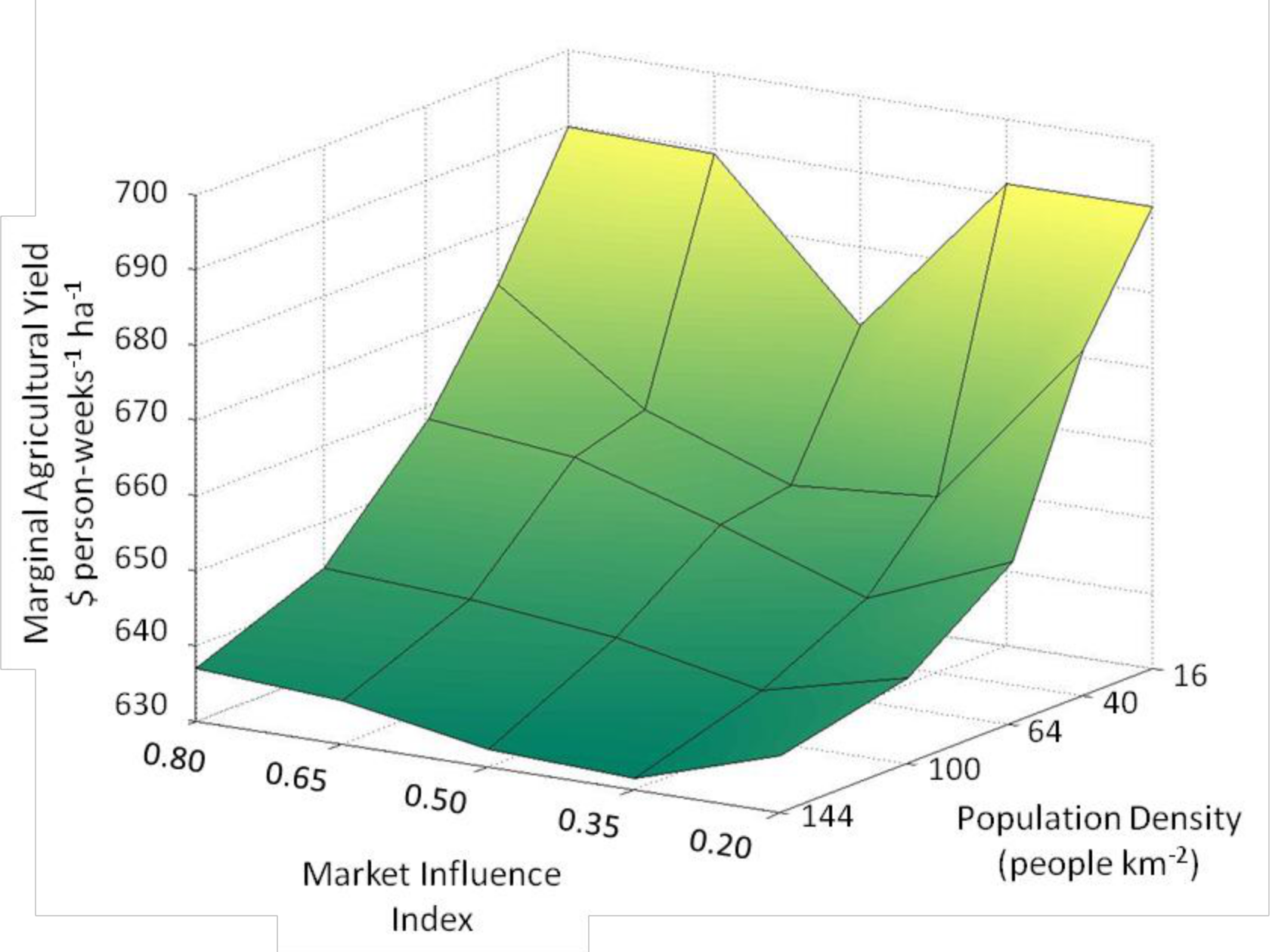

Supplement: Figure S3 — Marginal agricultural yield (per unit labor time and land) in response to varied market influence and population density levels with baseline environmental conditions. (TIF) [file pone.0073241.s003.tif]

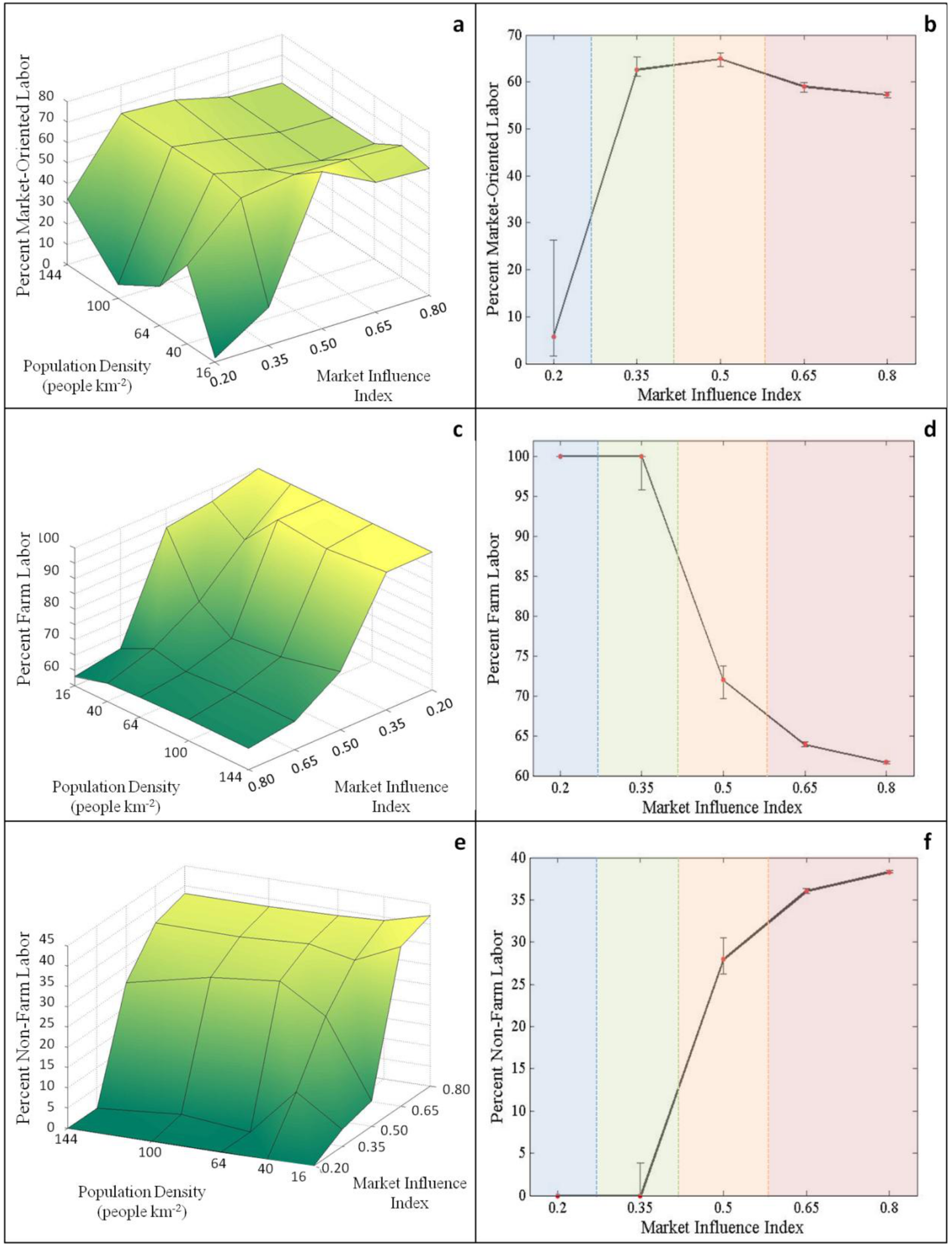

Supplement: Figure S4 — (a) Percent labor allocation toward market-oriented livelihood activities, (c) on-farm livelihood activities, and (d) non-farm livelihood activities in response to varied market influence and population density levels with baseline environmental conditions. Changes in labor allocation in response to shifts between livelihood strategies indicated by color-coded regions to Figure 4 (b, d, and f). (TIF) [file pone.0073241.s004.tif]

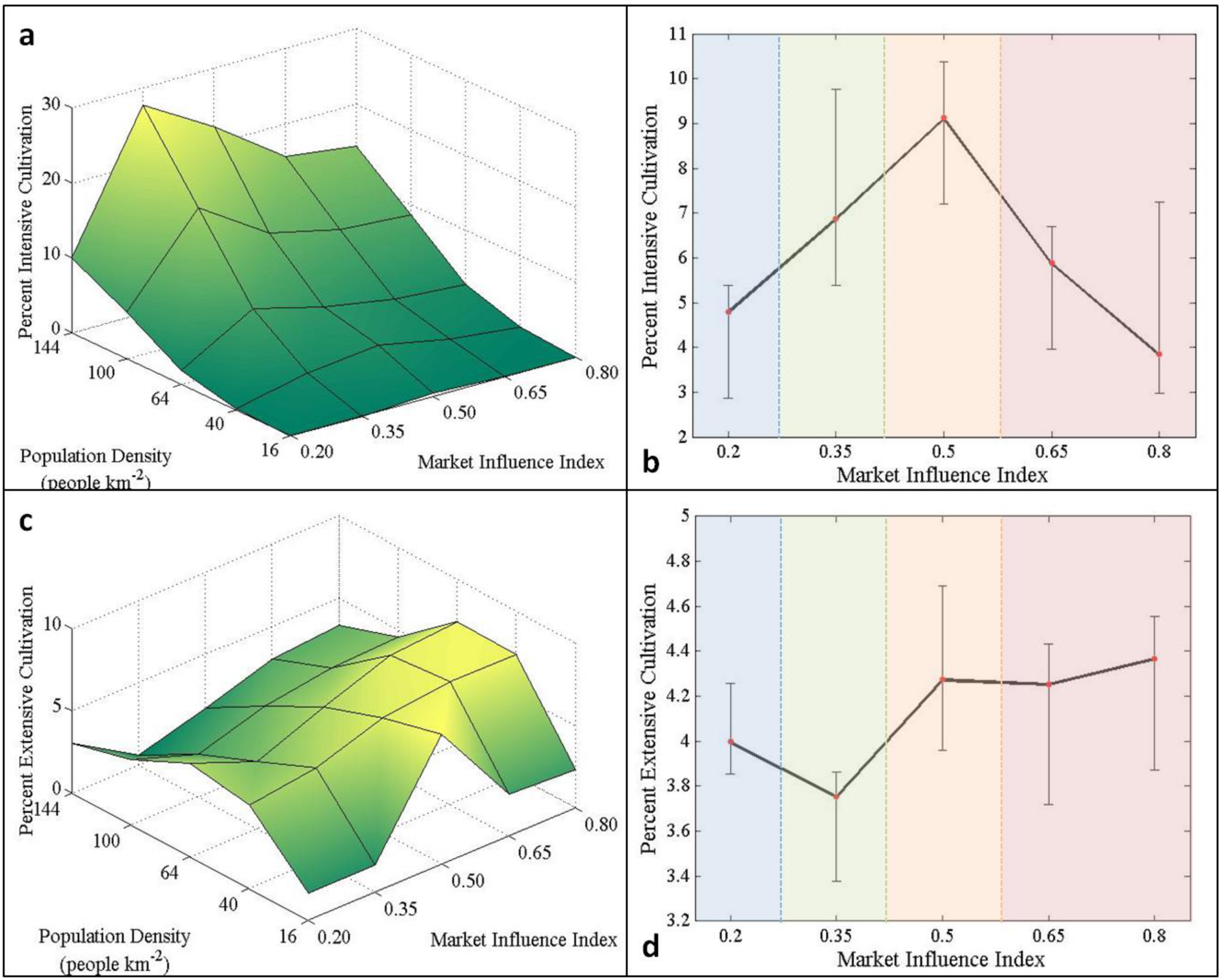

Supplement: Figure S5 — Percent intensive (a) and extensive (c) cultivation in response to variations in population density and market influence index settings from the baseline landscape. (b and d) Changes in percent intensive and extensive cultivation in response to shifts in livelihood strategies are indicated by color-coded regions according to Figure 4. (TIF) [file pone.0073241.s005.tif]
