# Supplementary material for: Exploring Agricultural Livelihood Transitions with an Agent-Based Virtual Laboratory: Global Forces to Local Decision-Making
Source: PLoS One. 2013 Sep 5;8(9):e73241. doi: 10.1371/journal.pone.0073241 (PMC3764159; doi:10.1371/journal.pone.0073241)
Supplement: Table S1 — Combined labor and input costs. (DOCX) [file pone.0073241.s006.docx]

**Table S1**: Combined labor and input costs.

|  | | **Land-Use *j*** | | | | | |
| --- | --- | --- | --- | --- | --- | --- | --- |
|  |  | **1** | **2** | **3** | **4** | **5** | **6** |
| **Land-Use *i*** | **1** | 4 | 3 | 2.5 | - | 0 | - |
|  | **2** | 4 | 2 | 2.5 | - | 0 | - |
|  | **3** | 4.5 | 3 | 1.5 | - | 0 | - |
|  | **4** | 8 | 5.5 | 4.5 | - | 0 | - |
|  | **5** | 7 | 2 | 4.5 | - | 0 | - |
|  | **6** | - | - | - | - | - | 0 |

Matrix of combined labor input costs, expressed in person-weeks ha^-1^, for converting from land-use *i* to *j*. Land-use are ‘intensive’ agriculture [1]; ‘extensive’ agriculture [2]; pasture [3]; forest [4]; fallow [5]; and dwellings [6]. Land-use conversions that are not possible (e.g. ‘intensive’ agriculture to forest) are indicated with ‘-‘ symbols. Based on Evans et al. (2001).
