# Supplementary material for: Exploring Agricultural Livelihood Transitions with an Agent-Based Virtual Laboratory: Global Forces to Local Decision-Making
Source: PLoS One. 2013 Sep 5;8(9):e73241. doi: 10.1371/journal.pone.0073241 (PMC3764159; doi:10.1371/journal.pone.0073241)
Supplement: Table S2 — Performance criteria associated with three agent-level behavioral patterns used to implement the genetic algorithm. (DOCX) [file pone.0073241.s007.docx]

**Table S2: Performance criteria associated with three agent-level behavioral patterns used to implement the genetic algorithm.**

| **Criteria** | **Threshold Value** | **Description** | **Source** |
| --- | --- | --- | --- |
| Normal Surplus | < 25% food surplus, at least 90% of time steps | Little or no surplus due to minimization of risk of and labor in agricultural production. | [18] |
| Minimum Aspiration Level | >= 90% of agents earn income >= farm costs (subsistence) or farm wage (market) | Income sufficient to support on-farm activities, or subjective income requirement. | [18] |
| Variance in Consumption | Coefficient of variation of consumption < 25% at least 90% of time steps | Livelihood diversification supports "consumption smoothing" between harvests. | [19] |
