# Supplementary material for: Exploring Agricultural Livelihood Transitions with an Agent-Based Virtual Laboratory: Global Forces to Local Decision-Making
Source: PLoS One. 2013 Sep 5;8(9):e73241. doi: 10.1371/journal.pone.0073241 (PMC3764159; doi:10.1371/journal.pone.0073241)
Supplement: File S1 — Supplementary Information. (DOCX) [file pone.0073241.s008.docx]

**Supplementary Information**

**ODD Protocol Model description**

*S1.1. Purpose*

This is a stylized agent-based model (ABM) of land-use and -cover change (LUCC) which encodes the decision rationale implied by induced intensification theory to provide mechanistic explanations of smallholder responses to changing economic, environmental, and demographic forces according to idiosyncratic risk preferences and environmental endowments. The model aims to reproduce the predicted relationship between population density and agricultural intensity as described by induced intensification theory. In addition, the model moves beyond the applicability of induced intensification theory by explicitly analyzing the decision-making processes of smallholding farmers leading to transitions to market-oriented livelihood activities.

Rather than attempting to recreate land-use patterns of specific systems, the aim is to develop a virtual laboratory in which hypotheses and model experiments are formulated to analyze the causes of smallholder behavior across a wide range of environmental and social conditions that are impossible to control and observe in the field. Beyond the specific theoretical insights addressed here, this agent-based model provides a virtual laboratory for testing the causal roles of demographic, economic and environmental factors in shaping a wide range of land-use systems and advances land-use theory in general.

*S1.2. Entities, state variables, and scales*

*S1.2.1. Agents*

Each agent represents a collection of smallholder households, the number of which varies with simulated population density, located in a single settlement that has 100 ha of land available for cultivation and settlement. Agent attributes are described in Table 1. Though most of the theory drawn upon conceives of the relevant decision making at the household level, a model of settlement agents is a reasonable approximation of the household context under the following assumptions: 1) households in the settlement are equally endowed with labor, land, and capital; 2) land-use choices are significantly constrained by land suitability; 3) settlement agents do not interact with one another; and 4) there are no significant spatial arrangements or interactions within the settlement that affect access to land. If all three assumptions hold, a model of household agents would produce identical results in terms of the areas allocated to each land-use activity, though the spatial patterns may be different. The settlement agent simplification is assumed so that population densities can be easily manipulated, which is critical to the model experiments. This formalization also does not require detailed knowledge of local land allocation mechanisms, thus maintaining the generality of model outcomes. Indeed, one or more of these assumptions are likely to be violated in real land systems, and the implications of the settlement area simplification will be discussed in light of the results obtained.

| **Attribute** | **Brief description** |
| --- | --- |
| *Population density* | The number of people allocated to a settlement area of 1 km^2^. |
| *Age structure* | The population is divided evenly between children and adults. Age structure is held constant and determines a settlement agent's labor supply and food and income demands. |
| *Household size* | All households are composed of two adults and two children, and aggregated to the settlement level according to population density. |
| *Stocks* | Initial food and income stocks are allocated based on minimum subsistence requirements. These are dynamically updated based on agents' land-use and livelihood decisions. |
| *Subsistence requirements* | Minimum subsistence requirements consist of grain for household food consumption and livestock feed [1]. Minimum monetary income requirements equal annual farm input costs plus the cost of a year’s worth of food should crops fail. An agent's minimum subsistence and income requirements equal those of an individual household multiplied by the number of households in the population. Children require half of the subsistence needs of an adult [2]. |
| *Labor supply* | Total available labor is expressed in person-weeks and is calculated by multiplying a year’s worth of labor net of required ‘home’ time (e.g. leisure, home maintenance, home textiles, etc.) by the size of the population [3]. |
| *Risk preferences* | A parameter ranging from 0 to 1, heterogeneous across agents, that weighs the potential pay-off of an activity against the certainty equivalent pay-off from a risk-neutral [4], [5]. Agents are assigned heterogeneous risk preferences drawn randomly from a normal distribution. |
| *Land-use preferences* | Agents are assigned uniform preferences across land-uses. However, future model versions can differentiate land-use preferences according to agricultural suitability, cultural preferences, or the relative contribution of each land use to the agent's income, for example. |
| *Subjective aspiration levels* | The wage rate of the livelihood activity forgone, which must be met or exceeded by the chosen livelihood activity (i.e. opportunity cost). |

*S1.2.2. Spatial Units*

Stylized landscapes of 100 by 100 square grids of cells are generated (Fig. 1), with each cell representing one hectare (total area = 100 km^2^). Each cell has a number of biophysical attributes.

| **Attribute** | **Brief description** |
| --- | --- |
| *Topography* | Percent slope is derived from a Digital Elevation Model (DEM). Slope is a proxy for soil suitability for agriculture [6]. |
| *Precipitation constraints* | Precipitation constraints are varied uniformly across the landscape as a proxy for number of growing days [7]. |
| *Agricultural suitability* | Slope and precipitation constraints impose zero to 100 percent reductions in agricultural yield according to agricultural suitability classes [6], [7]. |

Experimental landscapes are created by turning slope and/or precipitation constraints 'on' or 'off'.

| **Experimental Landscape** | **Brief description** |
| --- | --- |
| *Baseline* | Reduction in potential agricultural productivity due to slope constraints according to topography, and a 50 percent reduction due to precipitation constraints. |
| *Slope-Only Constrained* | Reduction in potential agricultural productivity due only to slope constraints according to topography. |
| *Precipitation-Only Constrained* | Reduction in potential agricultural productivity of 50 percent due only to precipitation constraints. |
| *Neutral* | No reductions in potential agricultural productivity. |

*S1.2.3. Land Uses*

Six different land uses are represented (Fig. 1b): three productive uses (intensive agriculture, extensive agriculture, and pasture for grazing livestock) and three non-productive uses (forest, fallow, and dwellings). ‘Intensive agriculture’ is defined as cultivation that uses external inputs (i.e. fertilizer, irrigation, and/or land improvement) to maintain productivity under repeated annual cultivation. Currently, the model is limited to single cropping, but future versions of the model will be extended to simulate multi-cropping cultivation systems. ‘Extensive agriculture’ is defined as cultivation with no external input, and is therefore subject to land degradation under repeated cultivation. ‘Pasture’ represents rangeland on which livestock grazing occurs, and is subject to degradation if grazed repeatedly without fallow. All agricultural yields are reported in grain equivalents (Table 1). Agricultural product prices are assumed equal to control for agricultural commodity-differentiated price effects, and are based on the 5-year average farm price of wheat [8].

| **Attribute** | **Brief description** |
| --- | --- |
| *Potential yield* | Crop or livestock yields per hectare (Table 1). |
| *Degradation rate* | Yields decline at varying rates depending on the type of land use (Table 1). |
| *Regeneration rate* | Yields recover during fallow periods after continual use at different rates depending on the type of land use (Table 1). |
| *Labor costs* | Labor costs varying according to the intensity of land-use, and are expressed in person-weeks per hectare (Table S1). |

*S1.2.4. Environment*

| **Attribute** | **Brief description** |
| --- | --- |
| *Population density* | Uniform across the landscape and expressed as people/km^2^ [9]. |
| *Market influence/access index* | The global/regional market setting of the focal landscape. Market influence determines relative farm-gate crop prices, farm input costs, non-farm wage rates, transportation costs to market, and non-farm employment transaction costs [10]. |

A set of cost and price functions are hypothesized that link global market influence index values to local farm-gate and food prices, farm input costs, and non-farm wages and transaction costs. Global commodity prices and U.S. minimum wage represent agricultural commodity prices and non-farm wages realized by a farmer in locations with a market influence index at or near one. Local product and factor prices and costs in locations with market influence less than one vary according to the cost functions below.

Farm-gate prices ($P_{j,t}$) for agricultural products produced by land-use *j* at time *t* are a function of mean agricultural commodity price (*P_0_*, Table 1), market influence index value (*MI*), and the crop price factor ($\beta_{pcrop}$).

$P_{j,t}=P_{0}*{MI}^{\beta_{pcrop}}$ [S-1]

The baseline non-farm wage rate (*W_o_*) is determined by the relationship between a benchmark non-farm wage (*NFW*; U.S. minimum wage at a MI of 1) and the global MI for the location subject to the non-farm wage factor (*β_nfwage_*).

$W_{o}=NFW*{MI}^{\beta_{nfwage}}$ [S-2]

Farm input costs (*C_farm_*) change proportionally with the baseline non-farm wage (*W_0_*) and market access (*MA*) at a rate determined by the farm cost factor (*β_fcost_*).

$C_{farm}=\beta_{fcost}\frac{W_{o}}{1+MA}$ [S-3]

If intensive cultivation is performed for sale on the market, labor time costs (*LC*) are converted to a monetary value to represent both the costs of non-labor inputs (e.g. fertilizer) and the opportunity cost of forgone non-farm wage labor.

Transaction costs associated with locating, securing, and maintaining non-farm wage employment (*C_nfarm_*) change proportionally with the baseline non-farm wage (*W_0_*) and market access (*MA*).

$C_{nfarm}=\beta_{nfcost}\left( 1-MA \right)W_{o}$ [S-4]

The effective non-farm labor wage rate is equal to the gross income net of transaction costs (Eq. S-4) per unit of labor time required to convert to or maintain in land use *j* from *i* (${LC}_{i\to j}$) which produces the given agents' most profitable agricultural commodity.

$W_{nfarm}=\left( W_{o}-C_{nfarm} \right){LC}_{i\to j}$ [S-5]

*S1.2.5. Spatial and Temporal Scales*

One model time step represents one year, and the model is run over a twenty-year period (with the first ten as model spin-up). The landscape is composed of 100 by 100 grid cells, each grid cell representing 1 ha, for a total area of 100 km^2^.

*S1.3. Process Overview and Scheduling*

The model uses a discrete event-sequencing framework (Fig. S2) in which each agent makes land-use decisions over their entire cultivation area sequentially, but the states of landscape cells across agents' holdings are updated synchronously. The main processes in operation each time step are biophysical regeneration/degradation, agents' labor allocation, land-use selection and harvest, and yield and price expectation formation for next period.

*S1.4. Design concepts*

*S1.4.1. Basic Principles*

A central theory explaining the dynamics of agricultural land use by smallholders is ‘induced intensification’ [11], which relates changes in farming systems to the behavioral responses of smallholders to dynamic demographic, economic, and technological factors. Early descriptions of agricultural intensification by Boserup [12] and Chayanov [13] described a process through which smallholders were forced to increase the labor-intensity of cultivation through techno-managerial innovations to meet increasing production demands from rising population density. A wide range of disciplines expanded on these insights to consider the roles of environmental suitability [14] and commercial agricultural activities [15]-[17] in driving agricultural intensification, which became more broadly labeled as 'induced intensification' theory [18].

This model attempts to enrich induced intensification theory by explicitly linking global market influence to land-use and livelihood decisions. The role of local economic conditions, especially non-farm wage opportunities in relation to land-use choices, has yet to be systematically linked to land-use intensity across locations globally. Applicable theory, however, has developed from the accumulation of case-study knowledge. de Janvry and colleagues [19] offer a generic explanation for variations in market participation across sites relating to local farm-gate prices, internal costs of production, and food prices. Missing or inefficient markets for agricultural products or input factors are commonly observed in agricultural systems in the developing world [4], [5], [19]. Such market failures occur when transaction costs are higher than potential gains, in which case non-market transactions (e.g. in-kind trade) may take the place of formal market transactions or transactions might fail to occur at all. According to [19], the potential for successful market transactions varies with particular households as a function of transport costs to and from the market, opportunity and transaction costs, and perceived risks associated with uncertain prices. A 'price band' results in which the sale prices of commodities, such as food and farm inputs, are fractions of their purchase prices [19]. The relationship between internal costs of production and farm-gate prices, which are dependent on local market influence (i.e. both physical access to markets and purchasing power), determine the value of agricultural products (i.e. shadow price) for a given household. The shadow price of agricultural products, relative to the costs of purchasing food on the market, structure the consumption and production decisions of households, and consequently their degree of market participation. If the shadow price of a given product or factor falls within the price band, it is more costly to acquire or sell it on the market than it is for the household to produce/consume it, thus no market transaction will occur.

The same logic applies to the relationship between farm and non-farm wages. Each of these factors is subject to both local labor market conditions and regional access to non-farm wage opportunities [4], [5], [19], [20]. Farm wages are influenced by access to the market, farm-gate prices, and the costs of agricultural inputs. Similarly, non-farm wages are influenced by the relative value of non-farm labor and transaction costs associated with locating, securing, and maintaining non-farm wage employment. When non-farm wage rates are above those obtained from on-farm labor, households may shift labor allocation away from the farm to include more non-farm activities. Thus, access to non-farm wage opportunities influences the intensity of land-use, as non-land-based income sources can supplement or fulfill food and income requirements [4], [5], [20]-[22]. Combined, these theoretical strands provide a potential framework for household consumption and production decision rules that explicitly link local economic conditions, household land-use decisions, and regional land-use outcomes.

The model is designed to reproduce the observed patterns of land-use in response to demographic, economic, and agro-ecological conditions. Agent-level behavioral rules are based on the theoretical frameworks of Boserup [12] and Chayanov [13], which provide a smallholder household rationale for cultivation choices in response to population pressure and labor and land constraints. However, Boserup and Chayanov stop at describing intensification of subsistence agriculture, and more recent literature describes the importance of further transitions within rural agriculture to market-based production. The 'livelihoods' perspective within the field of development economics provides a means for extending existing intensification theories by considering the role of market opportunities in agricultural production choices. The model's design incorporates livelihood diversification concepts [4], [19], [23], to explicitly represent non-farm wage opportunities and factors influencing agricultural production for the market. Integration of these household-level theoretical frameworks informs agents' behavioral rules for livelihood diversification, labor allocation, agricultural production mode, and land-use choices, and through the interaction of many agents with their environment, attempts to reproduce the system-level agricultural dynamics described by induced intensification theory from the bottom-up.

*S1.4.2. Emergence*

This model is designed to explore the decision-making processes of agents in response to varying demographic, economic, and environmental conditions and the land-use patterns that result. In addition, the livelihood choices of agents are analyzed with respect to the diversity and proficiency of labor allocation to on- and off-farm livelihood activities. Labor allocation arises from the decisions of individual agents based on their expectations of pay-offs from each livelihood activity, individual risk tolerances, and larger-scale demographic, economic, and environmental conditions. Although larger-scale factors influencing livelihood decisions are specified exogenously and held constant throughout a given simulation, agents learn to predict and adapt to dynamic local conditions. Livelihood choices are subject to some path-dependence and individual agents' learning abilities. Therefore, agents' final labor allocations and system-level land-use outcomes cannot be predicted from the model's initial conditions.

*S1.4.3. Adaptation*

Agents make livelihood and land-use decisions each period based on the success of past decisions and their expectations for pay-offs in the current period. Agents select the best livelihood activities and land-uses according their expected utilities, and can adapt to declining or improving yields from land-based activities resulting from past cultivation choices. Extensions of the current model could include additional sources of adaption. For example, agents could adapt their preferences for particular land-uses based on the proportion of revenue each produces.

*S1.4.4. Objectives*

Agents attempt to maximize expected utility in their livelihood and land-use decisions. Agents allocate labor to on- and non-farm activities proportionally to the ratio of expected wage rates. Land-uses choices are made cell-by-cell based on the highest expected utility among possible land-uses. Subsistence-oriented land-uses take precedent over market-oriented land-uses. Expected utility for subsistence land-uses is calculated as the marginal return per unit labor, and the best land-use is selected using a satisficing framework [23], [24]. Expected utility for market-oriented land-uses is calculated as the marginal return per unit labor net of production costs, and the best land-use is selected using a profit maximization framework.

*S1.4.5. Learning*

Agents have a set of prediction models for forming expectations of future yields and crop prices that they update each period as new information becomes available (see Section S1.4.6 below for description of the prediction models). The performance (i.e. error) of each model is tracked every period, and the agent acts on the prediction of the currently most successful model (i.e. the ‘active’ model). In the next period, actual yields and prices are realized and model performances are updated. Agents are therefore able to learn which models best predict yield and price trends, and can adaptively switch to following the predictions of a previously ‘dormant’ model if it out-performs the current ‘active’ model when conditions change.

*S1.4.6. Prediction*

Agents form expectations of agricultural yields and prices by detecting trends in past observations, which are extrapolated one period into the future to form expectations. Agents use a set of ‘backward-looking’ expectation models that have been adapted from their original use in financial agent-based markets [25], [26], to consider non-monetary and spatially explicit information. Each agent is randomly given a set of twenty prediction models that vary in the prediction method and time span over which past observations are considered. Each prediction model may use one of six different prediction methods that map past and present crop prices (P) and yields (given by substituting Y for P) into the next period using various extrapolation methods:

1. *Mean model*: predicts that *P(t+1)* will be the mean price of the last *x* periods.

$P\left(t+1\right)=\frac{\sum_{i=t-x:t} P\left(t_{i}\right)}{x}$ [S-6]

2. *Cycle model*: predicts that *P(t+1)* will be the same as *x* periods ago (cycle predictor).

$P\left(t+1\right)=P\left(t-x\right)$ [S-7]

3. *Projection model*: predicts that *P(t+1)* will be the least-squares, non-linear trend over the last *x* periods.

$P\left(t+1\right)=aP\left(t_{s}\right)^{2}+bP\left(t_{s}\right)+c$; [S-8]

where *t_s_* is the time span of *t-x* to *t­*, and *a*, *b*, and *c* are coefficients of fit.

4. *Mirror model*: predicts that *P(t+1)* will be a given fraction ξ of the difference in this period’s price, *P(t)*, from price *t-x* periods ago, *P(t-x)*, from the mirror image around half of *P(t)*.

[S-9]

5. *Re-scale model*: predicts that *P(t+1)* will be a given factor ζ of this *x* period’s price bounded by [0,2].

[S-10]

6. *Regional model*: predicts that *P(t+1)* is influenced by regional price information coming from neighboring agents.

The performance (i.e. error) of each model is tracked every period, and the agent acts on the prediction of the currently most successful model (i.e. the ‘active’ model). In the next period, actual yields and prices are realized and model performances are updated. Agents are therefore able to learn which models best predict yield and price trends, and can adaptively switch to following the predictions of a previously ‘dormant’ model if it out-performs the current ‘active’ model when conditions change.

*S1.4.7. Sensing*

Agents are assumed to know the suitability and potential yields of all possible land-uses on all cells within their cultivation area. Actual yields and prices are known only after agents engage in a particular land-use or livelihood activity. Agents keep a record of past yields and prices for all of their cultivated cells and livelihood activities, which is used to updated their prediction models.

*S1.4.8. Interaction*

Agents interact directly with the landscape through the selection of a cultivation method and corresponding land use. No interaction between agents exists. Future extensions of the model could include spatial interactions through land tenure rules and/or land markets, as well as the exchange of information and cultural norms through social networks.

*S1.4.9. Stochasticity*

Prediction methods and time horizons are randomly assigned among each agents' set of prediction models. No other sources of stochasticity currently exist. Extensions of the current model can explore the effects of stochasticity in crop prices and/or yields on agents' livelihood strategies and land-use choices.

*S1.4.10. Collectives*

Agents are themselves an aggregate representation of a number of individual households, which reasonably approximates the household context.

*S1.4.11. Observation*

Agent-level analysis was done by tracking the time path of labor allocation, production mode, and percent land cover for individual plots of agents 7 and 59 (Fig. S1), respectively, whose positions are indicated in Figure 1b, for the simulation with 64 people km^-2^. These agents were chosen because they had the same, roughly neutral risk preferences (0.53), yet they cultivated different quality land. Agent 7 was located on flat, highly productive land, whereas agent 59 was located on moderately sloped and initially partly forested land. The livelihood strategies of these agents differed accordingly. Agent 7 was able to maintain extensive cultivation for several periods longer than agent 59 due to higher agricultural productivity. Agent 59 was forced to switch to intensive cultivation early due to low and declining agricultural productivity in extensive cultivation. However, at a population density of 64 people km^-2^, both agents were eventually forced to adopt intensive cultivation to meet production demands.

*S1.5. Initialization*

Stylized landscapes of 100 by 100 square grids of cells are generated, with each cell representing one hectare (total area = 100 km^2^). The landscape is initialized with the highest potential productivity land uses according to agricultural suitability. Six different land uses are represented (Fig. 1b): three productive uses (intensive agriculture, extensive agriculture, and pasture for grazing livestock) and three non-productive uses (forest, fallow, and dwellings). Productive land uses are defined by functional group, rather than particular types (i.e. irrigated rice or shifting cultivation based on cassava), that vary in their potential productivity, degradation/regeneration rates (Table 1), and labor costs (Table S1).

One hundred agents are initialized in a evenly spaced grid across the landscape with 100 hectares (10 by 10) of land each. Each agent is randomly assigned a set of twenty prediction models that vary in the prediction method and time span over which past observations are considered (see Section S1.4.6). Risk preferences are assigned randomly from a normal distribution with mean of 0.5 ranging from one to zero. Initial food and income stocks are set to minimum subsistence levels for all agents.

*S1.6. Input Data*

Agents responded to constraints imposed and opportunities afforded by population density, environment, and market forces, which were represented by model relationships based on generalized empirical data for agricultural productivity, labor and transaction costs, agro-ecological dynamics, and a global index of market influence [10]. Input data used to parameterize agricultural productivities and biophysical processes or degradation and regeneration are described in Section S1.2.3. and Table 1. Labor costs for specific land-uses are adapted from case studies of land change and presented in Table S1. Local farm-gate and food prices, farm input costs, and non-farm wages and transaction costs in relation to the global market influence index are specified according to the procedure described in Magliocca and Ellis [27].

*S1.7. Submodels*

The main submodels include biophysical processes, yield and price expectation formation, expected utility calculation, labor allocation, and land-use selection. Yields from each land use are calculated for every landscape cell dependent on the time in the current land use and land-use-specific regeneration/degradation rates (Table 1). Expectation models are described in Section S1.4.6.

Agents derive utility from subsistence and monetary income. In this generalized context, income is defined as cash and food contributions to the welfare of the village derived from the set of livelihood activities in which village members are engaged. Utility from subsistence production follows a ‘satisficing’ framework and is derived as the marginal return from labor. In contrast, utility from market production follows a profit-maximizing framework and is calculated as marginal production net input costs.

For subsistence production, expected marginal utility is given by:

$EU(a,j)=\beta_{a,j}\frac{EY_{j,t}}{LC_{i\to j}}$; [S-11]

where the expected marginal utility from subsistence production of agent *a* for land-use *j* is the product of the *a*’s preference, β, for land-use *j* and the marginal return of expected yield, *EY*, at time *t* subject to labor costs, *LC*, of converting from land-use *i* to *j*. For market production, expected marginal utility is given by:

$EU(a,j)=\beta_{a,j}\left(EP_{j,t}EY_{j,t}-w_{f}LC_{i\to j}\right)$; [S-12]

where the expected marginal utility from market production is additionally a function of the expected price, *EP*, for production from land-use *i*, and the farm labor wage rate *w_f_*.

Labor allocation, expected utility calculation, and land-use selection are described as part of the model algorithm below.

The following algorithm describes the sequence of events for one simulated time period (year). The model is programmed in MATLAB. A decision tree of the labor allocation process is provided in Figure 2.

1. Agents determine the minimum amount of labor needed, $L_{sub}^{0}$*,* to meet minimum subsistence needs, $\delta_{\min}$, with the long-term average yield, *Y_j_^*^*, of the most productive land-use, *j*, on their land.

$L_{sub}^{0}=\frac{\delta_{\min}}{Y_{j}^{*}}$; [S-13]

1. Each agent calculates their risk-neutral expected returns, *ER_rn|a_*, of conservative activities (farm work and subsistence production) based on the discounted average observed yield and agricultural commodity prices (*P_i,t_*)of their most productive land use, farm wage (*W_farm_*), labor costs for maintaining land-use *j*.

${ER}_{rn|a}^{farm}=0.5\left( \frac{P_{j,t}Y_{j}^{*}}{W_{farm}{LC}_{i\to j}} \right)$; [S-14]

${ER}_{rn|a}^{sub}=0.5\left( P_{j,t}Y_{j}^{*}-W_{farm}{LC}_{i\to j} \right)$; [S-15]

1. Each agent calculates their risk-averse expected returns, *ER_ra|a_*, of conservative activities (non-farm work and market production) based on the expected yield (*EY_j,t_*) and agricultural commodity prices (*EP_i,t_*) of their most productive land-use, farm wage (*W_farm_*), non-farm wage (*W_nfarm_*), labor costs for maintaining land-use *j*, which are discounted by idiosyncratic risk preferences (*α_a_*).

${ER}_{ra|a}^{nfarm}=\alpha_{a}W_{nfarm}$; [S-16]

${ER}_{ra|a}^{mkt}=\alpha_{a}\left( {EP}_{j,t}{EY}_{j,t}-W_{farm}{LC}_{i\to j} \right)$; [S-17]

1. Based on the change in food (*S^food^*) and money (*S^mon^*) stocks, allocate proportion *λ_h_* of total labor (*L_TOT_*) to ‘home activities’ (*L_h_*).

$\lambda_{h}=1+\left(1-MI\right)\frac{\left(S_{a,t}^{food}-S_{a,t-1}^{food}\right)}{\delta_{\min}}+MI\frac{\left(S_{a,t}^{mon}-S_{a,t-1}^{mon}\right)}{\delta_{inc}}$; [S-18]

$L_{h}=\lambda_{h}L_{TOT}$; [S-19]

1. Based on risk-discounted expected returns from farm production (Eq. S-14) and non-farm labor (Eq. S-16), allocate labor to farm (*L_f_*) vs. non-farm (*L_nf_*).

$\lambda_{f}=\frac{{ER}_{rn|a}^{farm}}{{ER}_{ra|a}^{nfarm}}$; [S-20]

$L_{f}=\lambda_{f}L_{TOT}$; [S-21]

$L_{nf}=L_{TOT}-L_{h}-L_{f}$; [S-22]

1. Based on risk-discounted expected returns from subsistence (Eq. S-15) and market (Eq. S-17) production, allocate labor to subsistence (*L_sub_*) vs. market (*L_mkt_*) farm production.

$\lambda_{mkt}=\frac{ER_{ra|a}^{mkt}}{ER_{rn|a}^{sub}}$; [S-23]

$L_{mkt}=\lambda_{mkt}L_{f}$; [S-24]

$L_{sub}=L_{f}-L_{mkt}$; [S-25]

1. For all possible land uses in each of the cell in an agents’ landholdings, calculate expected marginal return on labor from subsistence production, and expected net marginal return from market production, and weight by land-use preferences to obtain expected marginal utility (Eqs. S-11 and S-12, respectively).
2. Agents first allocate subsistence labor (Eq. S-25) to cells that maximize marginal expected utility from subsistence production until subsistence labor or land constraints are met. Market labor (Eq. S-24) is allocated to remaining cells that maximize expected marginal utility from market production until market labor or land constraints are met.

**References**

1. Penning De Vries FWT, Rabbinge R, Groot JJR (1997) Potential and attainable food production and food security in different regions. Philos T R Soc Lond B 352: 917-928.
2. Evans T, Manire A, de Castro F, Brondizio E, McCracken S (2001) A dynamic model of household decision-making and parcel level landcover change in the eastern Amazon. Ecol Model 143: 95-113.
3. Macmillan W, Huang HQ (2008) An agent-based simulation model of a primitive agricultural society. Geoforum 39: 643-658.
4. Ellis F (1993) Peasant economics: farm households and agrarian development‎. Cambridge, UK: Cambridge UP.
5. Netting R (1993) Smallholders, householders: farm families and the ecology of intensive, sustainable agriculture. Palo Alto, CA USA: Standford UP.
6. Global Agro-Ecological Zones (GAEZ) (2011). Terrain Constraints. [online] URL: http://www.iiasa.ac.at/Research/LUC/GAEZ/index.htm. Accessed June 6, 2011.
7. Global Agro-Ecological Zones (GAEZ) (2011) Agro-climatic Constraints. [online] URL: http://www.iiasa.ac.at/Research/LUC/GAEZ/index.htm. Accessed June 6, 2011.
8. Schnepf RD (2008) High Agricultural Commodity Prices: What Are the Issues? CRS Report for Congress, Congressional Research Service, May 29, 2008.
9. Dobson JE, Bright EA, Coleman PR, Durfree RC, Worley BA (2000) LandScan: a global population database for estimating populations at risk. Photogramm Eng Rem S 66: 849–57.
10. Verburg PH, Ellis EC, Letourneau A (2011) A global assessment of market accessibility and market influence for global environmental change studies. Environ Res Lett 6. Available: http://iopscience.iop.org/1748-9326/6/3/034019/fulltext/.
11. Turner BL II, Hyden G, Kates RW (1993) Population growth and agricultural change in Africa. Gainesville, FL USA: University of Florida Press.
12. Boserup E (1965) The conditions of agricultural growth: the economics of agrarian change under population pressure. Chicago, IL USA: Aldine.
13. Chayanov AV (1966). In: Thorner D, Kerblay B, Smith, REF, editors. AV Chayanov on the theory of peasant economy. Homewood, IL USA: R. D. Irwin. pp 29–269.
14. Turner BL II, Hanham R, Portararo A (1977) Population pressure and agricultural intensity. Ann Assoc Am Geogr 67: 384-396.
15. Boserup E (1981) Population and technological change. Chicago, IL USA: University of Chicago Press.
16. Dorsey B (1999) Agricultural intensification, diversification, and commercial production among smallholder coffee growers in Central Kenya. Econ Geogr 75: 178-195.
17. Goldman A (1993) Agricultural innovation in three areas of Kenya: Neo-Boserupian theories and regional characterization. Econ Geogr 69: 44-71.
18. Turner BL II, Ali A (1996) Induced intensification: Agricultural change in Bangladesh with implications for Malthus and Boserup. Proc Nat Acad Sci USA 93: 14984-14991.
19. de Janvry A, Fafchamps M, Sadoulet E (1999) Peasant household behavior with missing markets: Some paradoxes explained. Econ J 101(409): 1400-1417.
20. Barrett C, Reardon T, Webb P (2001) Nonfarm income diversification and household livelihood strategies in rural Africa: concepts, dynamics, and policy implications. Food Pol 26: 315-331.
21. Chowdury RR (2010) Differentiation and concordance in smallholder land use strategies in southern Mexico's conservation frontier. Proc Nat Acad Sci USA 107 (13): 5780-5785.
22. Reardon T, Berdegue J, Barrett J, Samoulis K (2006) Household income diversification into rural nonfarm activities. In: Haggblade S, Hazell P, Reardon T, editors. Transforming the Rural Nonfarm Economy. Baltimore, MD USA: Johns Hopkins UP.
23. Brown D, Aspinall R, Bennett D (2006) Landscape Models and Explanation in Landscape Ecology—A Space for Generative Landscape Science? Prof Geogr 58: 369-382.
24. Schreinemachers P, Berger T (2006) Land use decisions in developing countries and their representation in multi-agent systems. J Land Use Sci 1: 29-44.
25. Arthur WB (1994) Inductive reasoning and bounded rationality. Am Econ Rev 84(2): 406-411.
26. Arthur WB, Durlauf S, Lane D (1997) The economy as an evolving complex system II. Sante Fe, NM USA: Addison-Wesley.
27. Magliocca NR, Ellis EC (2013) Using pattern-oriented modeling to cope with uncertainty in multi-scale agent-based models of land change. Trans GIS: DOI: 10.1111/tgis.12012.
